# New model of vincristine-induced neuropathic pain in children: a first step towards prediction and prevention

**DOI:** 10.1097/j.pain.0000000000000981

**Published:** 2017-06-22

**Authors:** Maria Fitzgerald

**Affiliations:** Department of Neuroscience, Physiology and Pharmacology, University College London, United Kingdom

It is an unfortunate fact of life that our understanding of paediatric neuropathic pain lags behind that of adults, despite clear evidence of clinical need.^5,7,14^ One important source of such pain is vincristine, a vinca alkaloid commonly used to treat paediatric cancers, which causes substantial neurotoxicity and neuropathic pain. Although peripheral neuropathy is a well-recognized side effect of vincristine therapy, the incidence, severity, and the long-term consequences of vincristine-induced neuropathy (VIPN) on the quality of life in children are not well understood.^10^ This knowledge gap is partly because of the lack of accepted and clinically feasible assessment tools in pediatric populations but is also because of the lack of a biological mechanistic understanding of how VIPN pain is generated and maintained in children.

The future is therefore substantially brighter following the publication of the first, well-defined, rat model of chemotherapy-induced neuropathic pain in early life. In this volume of *PAIN*,^12^ Schappacher, Styczynski, and Baccei use a careful systemic vincristine dosing regime in rats aged 10 to 21 days old to successfully balance the required neurotoxicity with the wellbeing of the rat pups and produce a convincing model of VIPN pain, measured as a significant and long-lasting behavioural mechanical hypersensitivity.

The model highlights some intriguing aspects of childhood chemotherapy pain. During the vincristine treatment regime itself, there was no effect upon mechanical sensitivity. Only 5 days after the treatment had ceased did the hypersensitivity emerge, equally in males and females, and continue well into adulthood. Thus consistent with classic models of direct nerve injury in young animals,^9,13^ the onset of VIPN hypersensitivity is delayed, emerging at adolescence (postnatal day 26). This differs markedly from the adult VIPN pain profile, where mice show significant mechanical hypersensitivity 24 hours after the first vincristine dose.^11^

The clinical symptoms of vincristine-induced peripheral neuropathy are often persistent and can sometimes worsen after the termination of chemotherapy or even emerge after drug withdrawal. There are a number of risk factors,^10^ including an inherited polymorphism in the promoter region of CEP72 associated with increased risk and severity of vincristine-related peripheral neuropathy,^3^ but one factor that shines through this and other studies is that peripheral neuropathic pain is age dependent.^4^ Following nerve trauma, the incidence of neuropathic pain in children increases with the age at which the nerve damage occurs^2^ and is not observed at all following newborn nerve trauma.^1^ Interestingly, increased childhood age is also associated with increased risk of VIPN pain,^3,8^ despite the same vincristine dosing limit at all ages.

One possible reason for this has been demonstrated in another rodent model of nerve injury, spared nerve injury, which at an early age triggers an active, constitutive, immune suppression of nociceptive pain activity in the spinal cord dorsal horn. In contrast to adult nerve injury, which evokes a proinflammatory immune response in the spinal dorsal horn, nerve injury at postnatal day 10 triggers an anti-inflammatory immune response, characterized by significant increases in IL-4 and IL-10^9^ and a shift in spinal cord microglia polarization to the M2 phenotype.^6^ As the nerve-injured mice reach adolescence (postnatal day 25-30), the dorsal horn immune profile switches from an anti-inflammatory to a proinflammatory response characterized by significant increases in tumor necrosis factor and brain derived neurotrophic factor^9^ and polarization of microglia to the M1 phenotype.^6^ This change in inflammatory profile is accompanied by late-onset neuropathic pain behaviour.

A causal link between the pain and the dorsal horn inflammatory profile is shown by the blockade of the anti-inflammatory activity with intrathecal anti-IL10 which unmasks neuropathic pain behaviour in young nerve-injured mice. This supports the existence of active pain suppression by a dominant anti-inflammatory neuroimmune response in the young central nervous system and raises the possibility that another cause of the prolonged VIPN pain in young patients with cancer may be the well-established immunosuppressive action of vincristine as well as direct nerve damage.

It is evident that more progress is needed to identify clinically useful predictors of VIPN and effective approaches for prevention or treatment of VIPN pain in both paediatric or adult patients. This new rodent model demonstrates that high-dose administration of vincristine during the postnatal period selectively evokes a mechanical hypersensitivity that is slow to emerge during adolescence, in marked contrast to the rapid-onset adult VIPN pain profile. This provides an excellent translational model for the special aspects of VIPN pain in children with cancer, which may emerge later, after treatment has ceased. It is a welcome step for a vulnerable chronic pain population.

## Conflict of interest statement

The author has no conflict of interest to declare.
